# Supplementary material for: Surface Microbiome Profiling of Dental Elevators Using Third‐Generation Sequencing: Implications for Infection Control in Dental Practice
Source: Microbiologyopen. 2025 Dec 8;14(6):e70178. doi: 10.1002/mbo3.70178 (PMC12685768; doi:10.1002/mbo3.70178)
Supplement: Supplementary file 1 — customer_backup. [file MBO3-14-e70178-s001.zip › customer_backup/customer_backup/function_analysis/BugBase/treat/otu_contributions/family/treat.family.Gram_Negative.pdf]

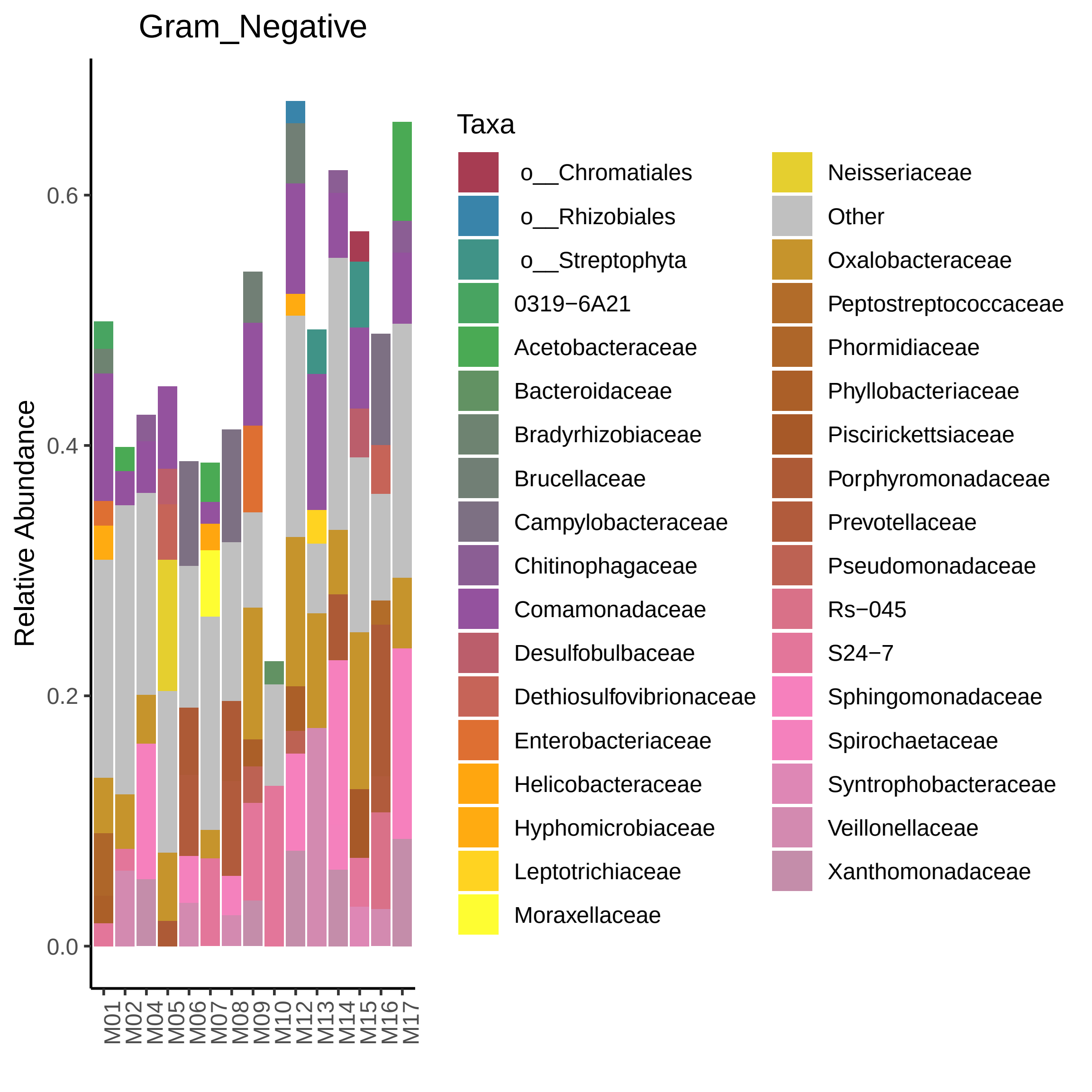
Gram_Negative
Relative Abundance
0.0
0.2
0.4
0.6
M01
M02
M04
M05
M06
M07
M08
M09
M10
M12
M13
M14
M15
M16
M17
Taxa
o__Chromatiales
o__Rhizobiales
o__Streptophyta
0319−6A21
Acetobacteraceae
Bacteroidaceae
Bradyrhizobiaceae
Brucellaceae
Campylobacteraceae
Chitinophagaceae
Comamonadaceae
Desulfobulbaceae
Dethiosulfovibrionaceae
Enterobacteriaceae
Helicobacteraceae
Hyphomicrobiaceae
Leptotrichiaceae
Moraxellaceae
Neisseriaceae
Other
Oxalobacteraceae
Peptostreptococcaceae
Phormidiaceae
Phyllobacteriaceae
Piscirickettsiaceae
Porphyromonadaceae
Prevotellaceae
Pseudomonadaceae
Rs−045
S24−7
Sphingomonadaceae
Spirochaetaceae
Syntrophobacteraceae
Veillonellaceae
Xanthomonadaceae
